# Lobar Hepatocellular Carcinoma with Ipsilateral Portal Vein Tumor Thrombosis Treated with Yttrium-90 Glass Microsphere Radioembolization: Preliminary Results

**DOI:** 10.1155/2013/827649

**Published:** 2013-02-17

**Authors:** M. Pracht, J. Edeline, L. Lenoir, M. Latournerie, H. Mesbah, O. Audrain, Y. Rolland, B. Clément, J. L. Raoul, E. Garin, E. Boucher

**Affiliations:** ^1^Comprehensive Cancer Center Eugène Marquis, CS 44229, 35042 Rennes, France; ^2^University of Rennes 1, 35043 Rennes, France; ^3^INSERM U-991 Liver Metabolisms and Cancer, 35033 Rennes, France; ^4^Department of Digestive Oncology, Comprehensive Cancer Center Paoli Calmettes, 13273 Marseille, France

## Abstract

Portal vein tumor thrombosis (PVTT) is a common complication of hepatocellular carcinoma (HCC) and has a negative impact on prognosis. This characteristic feature led to the rationale of the present trial designed to assess the efficacy and the safety of yttrium-90 glass-microsphere treatment for advanced-stage lobar HCC with ipsilateral PVTT. 18 patients with unresectable lobar HCC and ipsilateral PVTT were treated in our institution with ^90^Y-microS radioembolization. Patients were evaluated every 3 to 6 months for response, survival, and toxicity. Mean follow-up was 13.0 months (2.2–50.6). Outcomes were: complete response (*n* = 2), partial response (*n* = 13), stable disease (*n* = 1), and progressive disease (*n* = 2) giving a disease control rate of 88.9%. Four patients were downstaged. Treating lobar hepatocellular carcinoma with ipsilateral portal vein thrombosis with yttrium-90 glass-microsphere radioembolization is safe and efficacious. Further clinical trials are warranted to confirm these results and to compare ^90^Y-microS with sorafenib, taking into account not only survival but also the possibility of secondary surgery for putative curative intention after downstaging.

## 1. Introduction

Hepatocellular carcinoma (HCC) is a common cancer with an estimated annual incidence of 600,000 worldwide [1]. During the disease course, 40% of patients will develop portal vein tumor thrombosis (PVTT) [2, 3], a hallmark of advanced disease recognized as a poor prognosis factor by most classification systems: Barcelona Clinic Liver Cancer (BCLC) [4] and Cancer Liver Italian Program (CLIP) [5]. Patients with PVTT involving the portal trunk or main branch are considered to have advanced-stage disease (BCLC class C), independently of liver function or tumor size. Because of the risk of hepatic ischemia, the presence of PVTT complicates, or even contraindicates, locoregional treatments such as transarterial embolization/chemoembolization (TAE/TACE) designed to block arterial blood flow [6, 7]. For these patients with advanced-stage HCC, palliative systemic treatments may be proposed [8]: sorafenib is currently the gold standard, even if debated, allowing significant improvement in time to progression and survival [9].

Considering the arterial hypervascularization associated with PVTT [10] and the radiosensitivity of HCC [11], radioisotopes would be a logical therapeutic option. 

Recent retrospective analyses of single-center series have demonstrated an acceptable safety profile for new modalities of selective internal radiation therapy (SIRT) using radiolabeled glass microspheres, even in the presence of PVTT [12–18]. 

^90^Y-microspheres radioembolization is a recent concept in radiation therapy for HCC. Radiolabeled particles injected into the hepatic artery become trapped at the precapillary level where they emit potentially lethal internal radiation. This selective mechanism limits exposure to the surrounding normal parenchyma, thereby permitting higher dose delivery than with an external beam [14, 18]. 

 We report here our experience with 18 patients with lobar HCC and ipsilateral intrahepatic PVTT given SIRT using yttrium-90 glass microspheres (^90^Y-microS). These patients showing advanced diseases (PVTT) but involving only one lobe were treated with the intention to downstage their disease to a curative surgical treatment (transplantation or resection).

## 2. Materials and Methods

### 2.1. Patients and Tumors

From January 2007 to December 2010, 63 patients were treated in our center for HCC using intra-arterial ^90^Y-microS injections. Eighteen of these patients had lobar HCC with intrahepatic ipsilateral PVTT. All 18 patients had preserved hepatic function (<2.5XULN total bilirubine, <5XULN aminotransferases) and were in good general condition (WHO performance status score 0 or 1). 

This retrospective study group included twelve men and six women, mean age 64.4 years, age range 44–77 years (Table 1). Histological and radiographic diagnoses were established in 16 patients, radiographic diagnosis alone in two [19]. No attempt was made to obtain a biopsy of the portal thrombus prior to treatment, but triphasic helical computed tomography demonstrated contrast uptake kinetics characteristic of PVTT observed in HCC: arterial enhancement and portal washout. The pathology classification was severe fibrosis in two patients; the other 16 had cirrhosis. The main underlying etiology was alcohol (*n* = 10). All tumors were unilobular with 7 showing multifocal and 3 infiltrating patterns. The PVTT was ipsilateral in all cases, branch thrombosis in 10, and intrahepatic trunk thrombosis in 8, extending to lobular or segmental branches in 3 patients and to a hepatic vein in one. Serum alpha-fetoprotein level was below 400 ng/mL (parameter of the CLIP classification) in fourteen patients (normal in six), elevated in four (>400 ng/mL). For 13 patients, this protocol was the first-line treatment. Five patients had had one or more previous treatments: TACE (*n* = 3), sorafenib (*n* = 2), and radiofrequency (*n* = 2). Treatment with sorafenib was stopped at least one month before the SIRT procedure. One patient was treated with sorafenib during the procedure.

This retrospective study was approved by the institutional ethic board.

### 2.2. Treatment Planning

Pretreatment angiography and technetium-99 macroaggregated albumin single-photon emission computed tomography with CT coregistration (MAA SPECT/CT) were performed to assess gastrointestinal flow and lung shunting [20]. A glass-based device (TheraSphere, Ottawa, Canada) was used. The treatment by ^90^Y glass microspheres was carried out as described by Salem and Thurston [21]. The activity to be injected (*A*_inj_) was classically calculated with the aim of delivering a dose *D* of 120 ± 20 Gy to the volume to be treated (i.e., the injected liver, usually one lobe). This dose was calculated according to the following formula, based on the Medical Internal Radiation Dose (MIRD) formalism, previously described and widely used [12, 16, 21, 22]:

(1) $$\begin{array}{c} D_{\left(\text{Gy}\right)}\;=A_{\text{inj}(\text{GBq})}\cdot \left(1-S\right)\cdot 50/W_{\left(\text{Kg}\right)}, \end{array}$$

where “*S*” is the lung shunt fraction, and “*W*” is the weight of the injected liver.

### 2.3. Dosimetric Approach

SPECT acquisitions parameters were as follows: 32 projections, 180°, 128∗128, 30 s/projection (Symbia T2 gantry, Siemens, Germany). SPECT data were reconstructed using an iterative method (OSEM, 5 iterations, 8 subsets) with attenuation and scatter correction and then visualized with or without fusion with CT scan data.

The SPECT/CT quantitative uptake analysis of tumoral and nontumoral liver tissue was performed using the “volumetric analysis” software (Syngo workstation, Siemens). Briefly, this software allowed us to generate semiautomatically volume of interest (VOI) in the liver and tumor by means of an isocontour definition method. For each VOI, the threshold value was adjusted so that the isocontour of the distribution volume of MAA was superimposed on the fusion images that corresponded to the contours of the liver and tumor. These VOIs were then used to calculate the volumes of the liver and the tumor (expressed in mL) in addition to the total activity (expressed in counts) contained in the liver (CP_L_) and tumor (CP_tum_). Volume and total counts in the healthy liver (CP_HL_) were calculated by substracting liver and tumor parameters.

The dose absorbed in the tumour and in the healthy liver were then calculated using the classical formula:

*D*_(Gy)_ = *A*_(GBq)_ · 50/*W*_(Kg)_, using:

- the activity *A*_tum_ contained in the tumor (*A*_tum_ = *A*_inj(GBq)_ · (1 − *S*) · CP_tum_/(CP_tum_ + CP_HL_)) and its weight for the tumoral dose;
- the activity *A*_HL_ contained in the healthy liver (*A*_HL_ = *A*_inj(GBq)_ · (1 − *S*) · CP_HL_/(CP_HL_ + CP_tum_)) and its weight for the healthy liver dose.

### 2.4. Treatment Procedure

Within 2 weeks of the planning angiography study, the prescribed activity of ^90^Y glass microspheres was administered by placing the tip of the delivery catheter in the same anatomic position as that used for the 99mTc-MAA injection. Administration of ^90^Y glass microspheres was always done selectively, in a lobar or segmental manner. Bremsstrahlung imaging was performed to confirm pretreatment MAA SPECT data for tumor targeting.

### 2.5. Evaluation and Followup

Clinical and biological evaluations were done after 1 and 4 weeks and then every 3 months. Analysis of efficacy was done at 3 and 6 months and then every 3 to 6 months by assessing European Association for the Study of the Liver (EASL) response criteria [19]: complete response (CR): absence of any enhancing tissue; partial response (PR): >50% decrease in enhancing tissue; stable disease (SD): <50% decrease in enhancing tissue. Progressive disease (PD) was defined as any increase in enhancement of the treated tumor that clinically would translate into additional locoregional therapy (i.e., repeat ^90^Y). Safety was assessed according to the Common Terminology Criteria for Adverse Events CTCAEv3.0 criteria [23]. Pathologic analysis of resected specimens in secondly operated patients was performed.

### 2.6. Statistics

The database was closed on October 1, 2011, the date at which all data were censored. Progression-free survival (PFS) was measured from the date of the first treatment until the date of progression in the treated liver. Overall survival (OS) was measured from the date of the first treatment until the date of death from any cause or of last followup. SAS software was used to determine overall survival and progression-free survival using the Kaplan-Meier method. The Kruskal-Wallis test was used to look for differences between controlled and noncontrolled tumors and univariate analysis applied the chi-square test with *P* < 0.05 considered as statistically significant.

## 3. Results and Discussion

### 3.1. Patients and Treatments

The 18 patients received 21 treatments. Three patients received a second dose either because the first dosimetry was insufficient (*n* = 2; 101 and 106 Gy, resp.) or because of an insufficient target (*n* = 1 multifocal tumor with 2 feeding arteries). Treatments characteristics are shown in Table 2.

The pulmonary shunt, determined by monitoring albumin macroaggregates, was minimal (mean 6.0%, median 2.45%, range 0–43.5%) except in one patient (43.5% in a patient with a voluminous intratumor arterioportal shunt). Injected activity varied from 1.31 to 7.51 GBq (median 2.45 GBq). The lowest activity (1.31 GBq) was delivered for the patient with the intratumor shunt in order to hold lung exposure below 30 Gy (estimated 28.5 Gy). The median dose delivered to the target volume was 117.8 Gy, approaching the aim of 120 ± 20 Gy [12, 16, 21, 22]. Two patients received an intensification of the treatment (increased injected activity of 30 and 50%) regarding the large size of the lesions (10.6 and 13.0 cm, resp.), their necrotic character and the presence of recently described, favorable predictive dosimetric parameters (26). Twelve patients showed albumin macroaggregates in their PVTT on the pretreatment technetium-99 macroaggregated albumin scanning (MAA SPECT/CT).

Tolerance was good for all patients. Adverse effects were grade 3 or less on the CTCAEv3.0 scale. Three patients (16.6%) complained of abdominal, three (16.6%) developed asthenia, and one (5.5%) anorexia. Seven patients (38.9%) developed transient liver dysfunction: 6 decompensations with edema and ascite (including 1 with infection); 3 of the 6 were Child-Pugh B patientsand 1 hepatic encephalopathy. No deaths could be attributed to the treatment.

### 3.2. Response and Patient Survival Outcomes

Biological and radiological response assessments were accomplished at 3, 6, and 9 months for, respectively, 18, 17, and 14 patients.

Biological response was recorded in all 4 patients with an elevated AFP level (>400 ng/mL) with an AFP decline of more than 80%. Patients outcome are shown in Table 3.

**Figure 1:**
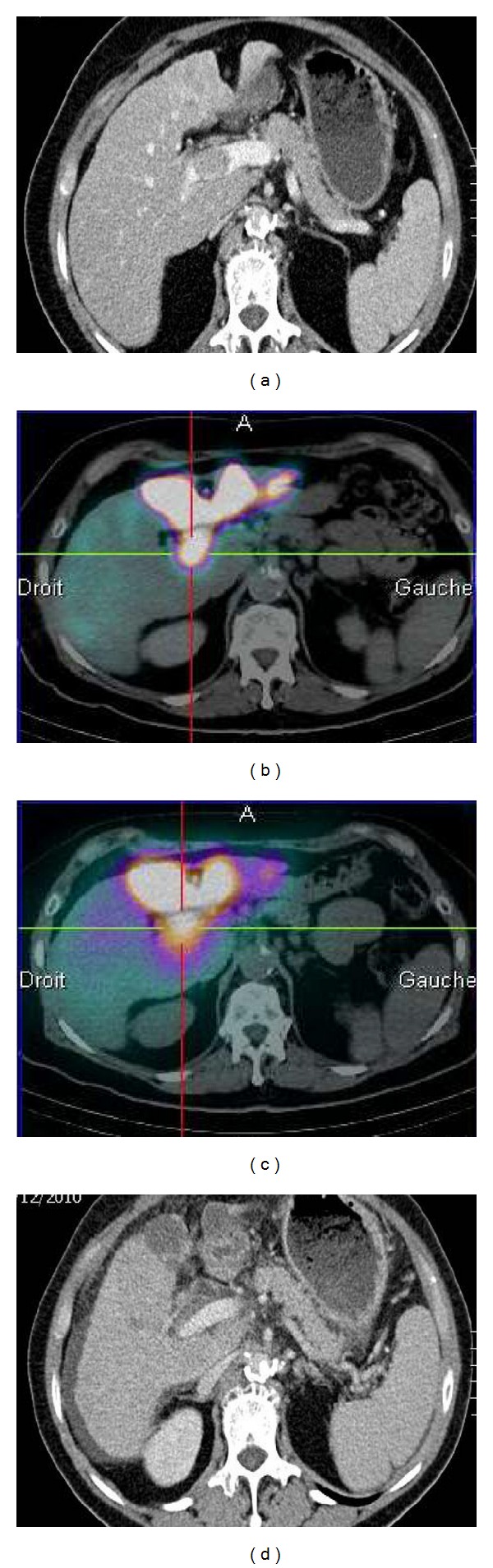
MAA SPECT/CT and CT scans showing implantation of microspheres in an PVTT and the involution of this PVTT after radioembolization: (a) Baseline CT scan, (b) MAA SPECT/CT showing a high MAA uptake in the PVTT, (c) Posttherapeutic Bremsstrahlung SPECT/CT after the injection of 4.5 GBq of 90Y-loaded glass microspheres: high uptake in the PVTT confirming the accurate implantation of microspheres in the PVTT, and (d) CT scan at 13 months.

 The disease control rate was 83.3% with 2 complete responses, 13 partial responses, and 1 stable disease. When considering these 16 controlled patients, 13 showed objective radiologic response for both tumor and PVTT. Partial patency of the portal vein was observed in eight patients and complete involution of the thrombus in three others. For 15 of the 18 patients (83.3%), there was a change in the aspect of the thrombus after treatment: objective decrease in contrast uptake due to devascularization of the portal tumor buds. Among the twelve patients with albumin macroaggregates deposit in their PVTT, nine showed patency of the portal venous network. Two of them had complete involution of their PVTT (Figure 1).

The two patients who received an intensified treatment showed a partial response without any significant toxicity.

**Figure 2:**
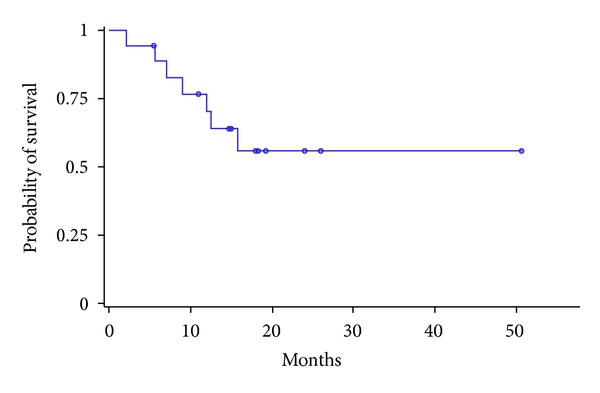
Kaplan-Meier analysis of overall survival (months): median overall survival not reached (95% CI, 9.0–∞) aften mean followup of 13.0 months.

**Figure 3:**
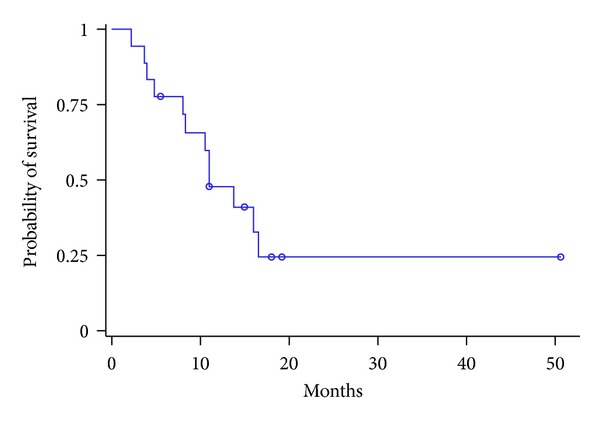
Kaplan-Meier analysis of progression-free survival (months): median progression-free survival was 11.0 months (95% CI: 8.0–16.5).

 Mean followup was 13.0 months (range 2.2–50.6). Followup was less than 1 year for six patients; four of them were dead. Survival rates at 6 months and 1 year were 88.5 ± 14.7% and 70.3 ± 21.1%, respectively. Median progression-free survival was 11.0 months (in the treated liver) after treatment end (95% CI: 8.0–16.5). Median overall survival was not reached (95% CI, 9.0–∞) (Figures 2 and 3). Seven patients (38.8%) had died at study end; deaths occurred 2.2 to 15.7 months after the first treatment. Four of these seven patients died because of the progression of the previously treated disease. 

At univariate analysis, there was a trend to significant difference (*P* = 0.07) between controlled and noncontrolled patients in term of median tumor absorbed dose (Table 2).

 Downstaging allowed surgery in four patients (22%). Two of them were downstaged to be within the transplantation criteria [19]; one had a liver transplantation but the second one refused it. They, respectively, showed a progression free survival and an overall survival of 50.6 and 50.6 and of 16.0 and 38.0 months. The two other patients did not meet the transplantation criteria but were eligible for surgical resection. One had a right hepatectomywhile the other was discovered a second HCC during the surgery that contraindicated the programmed hepatectomy. They respectively showed a progression free survival and an overall survival of 13.8 and 14.8 and of 10.5 and 12.5 months.

## 4. Discussion

PVTT is a poor prognostic factor for HCC. According to the BCLC classification [4], patients with PVT have advanced-stage disease, limiting options for surgery and intra-arterial therapy. Systemic administration of sorafenib can yield significant improvement in survival: median 8.1 months versus 4.9 with placebo for BCLC C patients with macrovascular invasion [26]. Sorafenib cannot, however, be considered as a curative treatment since there have been no reported cases of downsizing. The objective response rate with sorafenib (such as with other kinase inhibitors or monoclonal antibodies) is very low, less than 3% with the RECIST criteria.

Selective internal radiation therapy was developed in the 1990s for HCC with PVTT. ^90^Y is a higher energy beta emitter (Beta max 2.2 versus 0.6 MeV) with a broad cytoxicity range [27]. Radiation protection is a minor problem with ^90^Y, a pure beta emitter, allowing day hospital protocols. High activity doses may also be deliveredto the tumor: median 2.5 GBq in our study with a maximum of 7.5 GBq. The efficacy of these selective internal radiation treatments has not been assessed in prospective comparative trials (versus TACE, for instance), but generally is considered comparable, with better tolerance for radioembolization [28]. Data published to date on the use of radioembolization for palliative purposes in HCC patients with PVTT have demonstrated a very high response rate (50–70%) [29], especially if the new response criteria taking into account tumor vascularization are applied [30]. The safety profile appears to be similar to that observed in patients without PVTT, especially for the embolic risk. This is also the case for resin microspheres: a recent series had no cases of postembolization syndrome or of hepatic ischemia [13]. The expression “selective internal radiation therapy” should be preferred to the term “radioembolization” since the embolic effect is marginal so that microspheres are not to be contraindicated in patients with PVTT.

Our series confirmed the safety of this treatment, a further argument favoring its efficacy on the thrombosis. Using the EASL criteria, the tumor was controlled in 88.9% of our patients at three months [19]; the thrombosis regressed in the majority (11/18). This antitumor effect allowed downstaging to surgery in four patients (22%). Pathology examination of the explant in the patient who underwent liver transplantation showed a major histological response in the tumor nodules (the largest exhibited a totally necrotic aspect). The survival rate (88.5% at six months) was promising, even better than with sorafenib: mPFS = 11.0 in our series versus 4.1 months in the subgroup with macrovascular invasion of the SHARP trial [26]. Our results appear a little better than those previously published (Table 4) probably because this cohort is homogenous, containing only patients with lobar HCC and intrahepatic ipsilateral PVTT whereas patients in previously published studies showed indifferently lobar or bilobar HCC and extended PVTT (extrahepatic or bilateral). These results are, however, in agreement with those just published in a prospective phase 2 by Mazzaferro et al. [25] showing a disease control rate of 74.3%, an mPFS of 7.0 (6.0–12.0), and an mOS of 13.0 (9.0–17.0) months.

Like for some of these studies, our results confirm that downstaging advanced HCC with PVTT is not an unrealistic objective. Indeed, allowing to 22% (4/18) of these patients a downstaging to a curative surgical stage is a very encouraging data in the context of the classical poor outcome of this disease. Concerning the 14 remaining patients, 11 showed an objective response but unfortunately insufficient for a secondary curative surgery (insufficient tumor size reduction and/or incomplete involution of the PVTT). Only 3 patients did not respond to the treatment. For all 3, ^90^Y glass microspheres were a front-line treatment and their median TD was acceptable, higher than 114 Gy. Two of these failures could be explained by tumor sizes higher than 10 cm and the last one could be due to an underlying advanced cirrhosis (Child-Pugh B7), conditions known to be less favorable for SIRT.

Our interesting results do not seem influenced by the 27.7% (5/18) of patients previously treated. Indeed, even if all these patients showed a partial response on their liver tumor, 2 only of them showed partial patency of their portal vein tumor thrombosis. On the contrary, all these 5 patients showed adverse events (ascite), underscoring the risk of worse tolerance in case of pretreated tumors, especially after chemoembolization or large liver resections. 

 Regarding tumoral dosimetry, this study brings an interesting result as controlled lesions had higher dosimetry with a median dose to the tumor 284.5 Gy (194–393.9). However, the median tumor absorbed dose cannot in this small cohort be hold as a predictive factor of tumor response despite a trend to significance (*P* = 0.07) probably because a lack of power. This point needs to be highlighted in the next future as MAA SPECT/CT-based dosimetry results are available after the diagnostic angiography and before treatment and we will further explore it by continuing publishing the data of our patients showing lobar HCC with ipsilateral PVVT. It seems that to achieve an objective response, a sufficient activity of ^90^Y-microspheres needs to be injected. This kind of dose/response correlation has already been described by Ho et al. in 1996 [22] in the classical partition model. In this model, tumoral dosimetry (and tumoral activity) was evaluated using a tumor-to-nontumor uptake ratio evaluated by SPECT and not a direct calculation of the tumoral activity as we proposed here. The accuracy of this partitional model was not optimal (37.5% of response if the tumoral dose was higher than 225 Gy and 10.3% if it was below this value) and tumoral dosimetry has not yet been proposed for the treatment planning (i.e., for the calculation of the activity to be injected). Our results may change this point in the next future. Indeed, we recently published preliminary results on 36 HCC patients confirming this close dose response correlation [31]: a threshold dose of 205 Gy was predictive of the response with a sensitivity of 100%, a specificity of 66%, and an accuracy of 91% (only 3 nonresponders above 205 Gy on 36). Another confirmation has been brought by Chiesa et al. [32] who founded a median dose of the responding lesion of 431 Gy as against only 199 Gy for the nonresponding lesion (*P* < 0.0001). The tumoral dosimetry may now be shown not only as a part of the treatment planning but also as a tool to optimize the effectiveness of ^90^Y-microspheres radioembolization.

 One of the major drawbacks of the present study is its retrospective design and the lack of control group. Systemic therapy with sorafenib was not started at the same time after ^90^Y treatment in all patients who showed progressive disease, and this may have influenced overall survival. Finally, the small number of patients did not allow the calculation of significant difference in median tumor absorbed dose between controlled and noncontrolled tumors.

Based on the results of the present study, it appears that ^90^Y radioembolization in patients with unilobar HCC and an ipsilateral intrahepatic PVTT confirmed the safety and antitumor efficacy of this therapeutic option in this population with an unfavorable prognosis with more than 20% of patients downstaged to a curative approach. Moreover, this study hypothesized a predictive value of the MAA SPECT/CT-based dosimetry on tumor response. Dosimetric optimizations could lead in the future to significant improvements of ^90^Y radioembolization efficacy. 

In light of our findings, a randomized trial would be useful in this population to compare ^90^Y radioembolization versus sorafenib alone. In addition to overall survival, the trial should consider secondary curative surgery after downsizing as a main outcome criterion, similar to studies on liver metastases from colorectal adenocarcinoma [33]. There should, however, be some limits on inclusion: lobar HCC, ipsilateral branch or intrahepatic trunk PVTT and an appropriate liver function. Patients with bilateral thrombi or extension to the extrahepatic trunk would be poor indications due to the unfavorable benefit-risk ratio. 

## Figures and Tables

**Table 1:** Baseline characteristics (*n* = 18).

Characteristic	Value
Mean/median age (range) (y)	64.4/63.0 (44–77)
Sex ratio M/F (*n*)	12/6
Cirrhosis/fibrosis (*n*)	16/2
Main etiology (*n*):	
Alcohol/HCV/haemochromatosis/ dysmetabolic syndrome	10/4/3/1
Child-Pugh score (*n*): A5-6/B7	13/5
CLIP score (*n*): 2/3/4	11/5/2
Distribution (*n*): unifocal/multifocal/infiltrating	8/7/3
Mean/median (range) size (cm)	8/8.75 (3–13)
Portal vein tumour thrombosis (*n*):	
Branch	10 (8 right/2 left)
Trunk (or trunk + branch)	8
Tumour location (*n*): right/left	12/6
Mean/median (range): alpha-fetoprotein (ng/mL)	7932/36.5 (3–91000)

**Table 2:** Treatment characteristics.

Characteristic	Value
Selectivity of 90Y injection: whole liver/lobar/segmental	0/17/1
Median (range) activity (GBq)	2.5 (1.31–7.51)
Median (range) dose to the target volume (Gy)	117.8 (80.1–164.1)
Median (range) dose to the nontumoral liver (Gy)	75.5 (26.5–114.5)
Median (range) dose to the lungs (Gy)	3.8 (0–28.5)
Median (range) dose to the tumour (Gy)	261.1 (114.5–393.9)
Median (range) pulmonary shunt fraction (%)	2.4 (0–43.5)
Median (range) dose of controlled patients (complete response + partial response + stable disease)	284.5 (194.0–393.9)
Median (range) dose of nonresponders patients	181.5 (114.5–248.6)

**Table 3:** Treatment outcomes (*n* = 18).

Characteristics	Value
Dead/alive (*n*)	7/11
Response (*n*) CR/PR/SD/PD	2/13/1/2
Objective response/disease control rate (%)	83.3/88.9%
Median (95% CI) time to progression (months)	11.0 (8.0–16.5)
Median (95% CI) overall survival (from date of treatment)	Not reached (9.0–∞)
Downstaging to surgery or transplantation criteria (*n*)	4/18
Overall survival at 6 months (% and 95% CI)	88.5 ± 14.7
Overall survival at 1 year (% and 95% CI)	70.3 ± 21.1

**Table 4:** Published studies on the treatment of HCC with PVTT with yttrium-90.

Study (author/year/microspheres type/reference)	PVTT population/total population	6-month survival (%)	Disease Control rate at *X* months (%)	Median OS (months)	Median PFS (months)
Salem et al., 2004: glass [15]	*n* = 15/15	53	—	7.1 (4.1–13.9)	—
Woodall et al., 2009: glass [18]	*n* = 15/52	—	—	3.2	—
Kulik et al., 2008: glass [14]	*n* = 37/108	—	—	*Branch* PVTT: 9.9 (7.1–15.7) *Main* PVTT: 4.4 (2.9–7.4)	—
Tsai et al., 2010: glass and resin [17]	*n* = 22/22 15 evaluable	—	2-3 months: 58 (RECIST)	7.0	—
Iñarrairaegui et al., 2010: resin [13]	*n* = 25/25	64	2 months: 66.7 and 6 months: 50 (RECIST)	10.0 (6.6–13.3)	—
Hilgard et al., 2010: glass [12]	*n* = 33/108	65 (95% CI 46–92%)	3 months: 90 (RECIST) 94 (EASL)	10.0 (6.0–∞)	8.0 (5.9–∞)
Salem et al., 2010: glass [16]	*n* = 92/291 (35 *Child A* and 57 *Child B*)	—	2-3 months: 50 *Child A* and 32 *Child B *(EASL)	10.4 (7.2–16.6) *Child A* 5.6 (4.5–6.7) *Child B *	5.6 (2.3–7.6) *Child A* 5.9 (4.2–7.9) *Child B *
Sangro et al., 2011: resin [24]	*n* = 76/325 (branch + main)	—	3 months: 88.9% (EASL)	10.2 (7.7–11.8)	—
Mazzaferro et al., 2012: glass [25]	*n* = 35/52 (branch + main)	—	3 months: 74.3 (EASL)	13.0 (9.0–17.0)	7.0 (6.0–12.0)
